# The Use of Deep Learning and VR Technology in Film and Television Production From the Perspective of Audience Psychology

**DOI:** 10.3389/fpsyg.2021.634993

**Published:** 2021-03-18

**Authors:** Yangfan Tong, Weiran Cao, Qian Sun, Dong Chen

**Affiliations:** ^1^School of Art, Wuhan University, Wuhan, China; ^2^College of Network Communication, Zhejiang Yuexiu University of Foreign Languages, Shaoxing, China; ^3^School of Economics and Management, Southwest Petroleum University, Chengdu, China; ^4^College of Business, Gachon University, Seoul, South Korea

**Keywords:** AI technology, convolutional neural network, film and TV production, Computer Graphics, human-computer interaction

## Abstract

As the development of artificial intelligence (AI) technology, the deep-learning (DL)-based Virtual Reality (VR) technology, and DL technology are applied in human-computer interaction (HCI), and their impacts on modern film and TV works production and audience psychology are analyzed. In film and TV production, audiences have a higher demand for the verisimilitude and immersion of the works, especially in film production. Based on this, a 2D image recognition system for human body motions and a 3D recognition system for human body motions based on the convolutional neural network (CNN) algorithm of DL are proposed, and an analysis framework is established. The proposed systems are simulated on practical and professional datasets, respectively. The results show that the algorithm's computing performance in 2D image recognition is 7–9 times higher than that of the Open Pose method. It runs at 44.3 ms in 3D motion recognition, significantly lower than the Open Pose method's 794.5 and 138.7 ms. Although the detection accuracy has dropped by 2.4%, it is more efficient and convenient without limitations of scenarios in practical applications. The AI-based VR and DL enriches and expands the role and application of computer graphics in film and TV production using HCI technology theoretically and practically.

## Introduction

In recent years, as artificial intelligence (AI) technology develops continuously, algorithms and technologies based on deep learning (DL) have gradually permeated various aspects of daily life, and film and TV production are one of them (Lyczba, 2019). Films and television, especially films, are closely related to technologies. Films are born out of a technological revolution, and the developments of each technology bring changes to the film production industry (Kim, 2019). People are suffering from incredible pressure in modern society, and they walk into the cinema for the vent of emotions and audiovisual enjoyment. Film and TV special effects technologies are an effective means to create these audiovisual effects (Chen, 2019). The reality reflected by film and TV special effects is visual psychological reality.

As an industrial production, a means of communication, and an art form, films have always depended on technological innovation. The technology's application in films plays an important role in films' expressions. The pictures' movements and the images' modeling are significant characteristics of the films. Films can be more attractive through excellent picture movements and image modeling (Belyaev et al., 2020). A film is an art form subject to mass media, and its visual appreciation can satisfy and comfort the audiences (Matbouly, 2020). Therefore, better usage of technologies in modeling and movements provides the audiences with a better experience and psychological feelings, so that the audiences look forward to the visual effects and scenes (Schmlzle and Grall, 2020). Meanwhile, how to better apply AI technology, especially the technology related to film and TV production, to bring more exciting and lifelike experience to the audience is also widely concerned (Abbasi and Jamei, 2019). Communication technology is also one of the critical contents in films and TV interface design. Human-computer interaction (HCI) technology provides numerous information and prompts through output or display devices. In turn, people will feed back relevant information, answers to the questions, and prompts to the computer through input devices. However, there are still some shortcomings in the real-time performance of the existing technologies, and the technical solutions that can meet the needs of multiperson, real-time, and 3D attitude data output at the same time still need to be studied. Meanwhile, there is also a lack of complete technical solutions of acquisition, analysis, output, and operation. Therefore, a series of technical solutions based on neural network structure to realize real-time human posture recognition and runtime output are proposed, which have better performance than traditional technologies for real-time, multiperson, 3D posture recognition and other requirements.

This exploration aims to study the application of virtual reality (VR) technology and DL-based technology in the field of HCI, the significance of the technology to the production of modern film and television works, and its impact on the audience's psychology under the development of AI technology. In film and TV production, audiences have a higher demand for verisimilitude and immersion of the works, especially in film production. Based on this, a 2D image recognition system and a 3D recognition system for human body motions based on the convolutional neural network (CNN) algorithm of DL are proposed; an analysis framework is constructed. The proposed systems are simulated on practical and professional datasets, respectively.

## Methods

### Film and TV Production and Audience Psychological Changes With HCI Technology

In the AI technology era, VR and DL technologies based on DL have gradually permeated the film and TV industry, especially the film production industry (Nauryzbaev and Shomanova, 2018). Today's films have undergone dramatic changes from contents to forms. Such changes are brought about by the development of human society and technology, which are also products of the continuous changes in human aesthetic needs and audience psychology (Zhu, 2018). Digital technology breaks the restoration and shaping of the real world in film and TV operation, especially in film production. With brand-new AI technology, scenes and objects that do not exist in the real world can be created in films; images of different times and spaces in the real world can be combined, and real images and the illusory images can even be juxtaposed. Therefore, the audiences are immersed and enjoy the unprecedented audiovisual experience by integrating senses such as vision, hearing, gustation, and touch (Raney et al., 2018). Nowadays, human-computer interactive films, known as third-generation films, are also quietly changing the film industry. HCI is the most crucial factor that reflects the relationship between humans and technology. It makes the most sophisticated intelligent body, human, and the computer's automatic control system form a larger self-feedback loop. The audiences can experience the scenes and images in the movie through increasingly realistic virtual images. They can have a continuous sense of movement watching a single still image, which is also inseparable from audiences' psychological activities (Rogers, 2020). Except for the audiences' physiological “persistence of vision,” the motion on the screen is originated from the fabrication of the audiences' psychological activities. Immersion glasses based on VR combined with multimode HCI improve the user's perception through VR glasses and multimode technology. Moving pictures are organized through the still pictures with personal thoughts, thus forming the cognitive experience of the “moving screen.” With psychological influences, the audiences fully recognize and accept this false movement (Hamilton-Webb et al., 2019).

Research on the audiences' thinking modes and social life background reveals that their lives are inseparable from digital technologies in many aspects in the 21st century and full of AI-, VR-, and HCI-based products, which bring various convenient entertainment for them (Gruenewald and Witteborn, 2020). After digital technology is introduced to film production, boasting virtual effects in the illusory space fully meet the audiences' aesthetic expectations and needs in film language and picture effects (Harkema and André, 2020). Visual impacts are the most substantial aesthetic feelings that digital special effects bring to the audiences, which is a significant source of the film's visual aesthetics (Bramley et al., 2018). The 3D images' presentation, the digital Dolby sound system, and the VR technology's application have broken the pure visual appreciation limitations, and “watching” films becomes a multisensory aesthetic perception (Wu et al., 2019, 2020). In the dimension of film and TV production, animated film is taken as an example. The traditional animation production mode mostly uses the centralized way to make and render. After the emergence of 6G DL technology, it can achieve a high degree of computing and storage, which makes distributed cross-domain collaborative production possible. For example, DL +4k/8k shooting will realize real-time shooting, real-time transmission, cloud rendering, and cloud production. It achieves remote processing and multipoint coproduction, and effectively improves the efficiency and effect of film shooting; DL +VR/AR fully meets the new service requirements of the film and TV entertainment industry.

The new way of emotional experience involves almost all human sensory functions. The “concretization” of thinking, emotions, sounds, visions, touches, and inductions in the films advances the original experience to an all-around experience, which is more authentic and profound (Colonnese and Phillips, 2018). The audiences in traditional film-watching feel alienated from the films due to the alienation 2D screen brings. However, the existing VR imaging technology's verisimilitude closes the gap between the subjects and the traditional objects in film watching. In 3D film production, the torus space is continually advancing toward realism in the virtual environment (Changwook et al., 2018). The AI-based DL algorithms' application and practice in film and TV production are analyzed, and a 3D film and TV production method based on CNN is proposed.

### Human Action Recognition in 2D Images Based on CNN of DL

Many film companies have adopted schemes based on visual markers or sensors. The actors/actresses wear special clothing with visual markers or sensors. Then, a unique system is used to capture the positions of the human body's joints, thereby realizing human motion capture (Rogoza et al., 2018). Each camera estimates the human body motion in the 2D image in the current shooting frame according to the visual markers; next, the estimation results of multiple cameras are combined to generate the 3D motion data. Thus, the corresponding virtual character's actions are generated according to the data collected.

However, this scheme cannot fully meet the application needs, and there are still many limitations in practice. One reason is the limited real-time performance (Lv et al., 2014, 2017). Only Open Pose's high-end GPN can meet the real-time requirements, while the low-end cannot (Eline and Reijne, 2018). Many 3D motion estimation technologies that claim to be real-time rely on high-quality 2D image motion input, which takes time (Saunders et al., 2019). The other reason is that there are currently no complete schemes for 2D image motion extraction, 3D motions, and estimation. Therefore, many films, games, and VR companies need complete and impeccable schemes (Nie et al., 2019).

A single camera's RGB image is input to the proposed 2D image motion detection framework. Positions of the key points and some auxiliary information for key point groupings are output through the original neural network structure. Final positions for the human body's key points in images are obtained through the post-processing algorithms.

**Figure 1:**
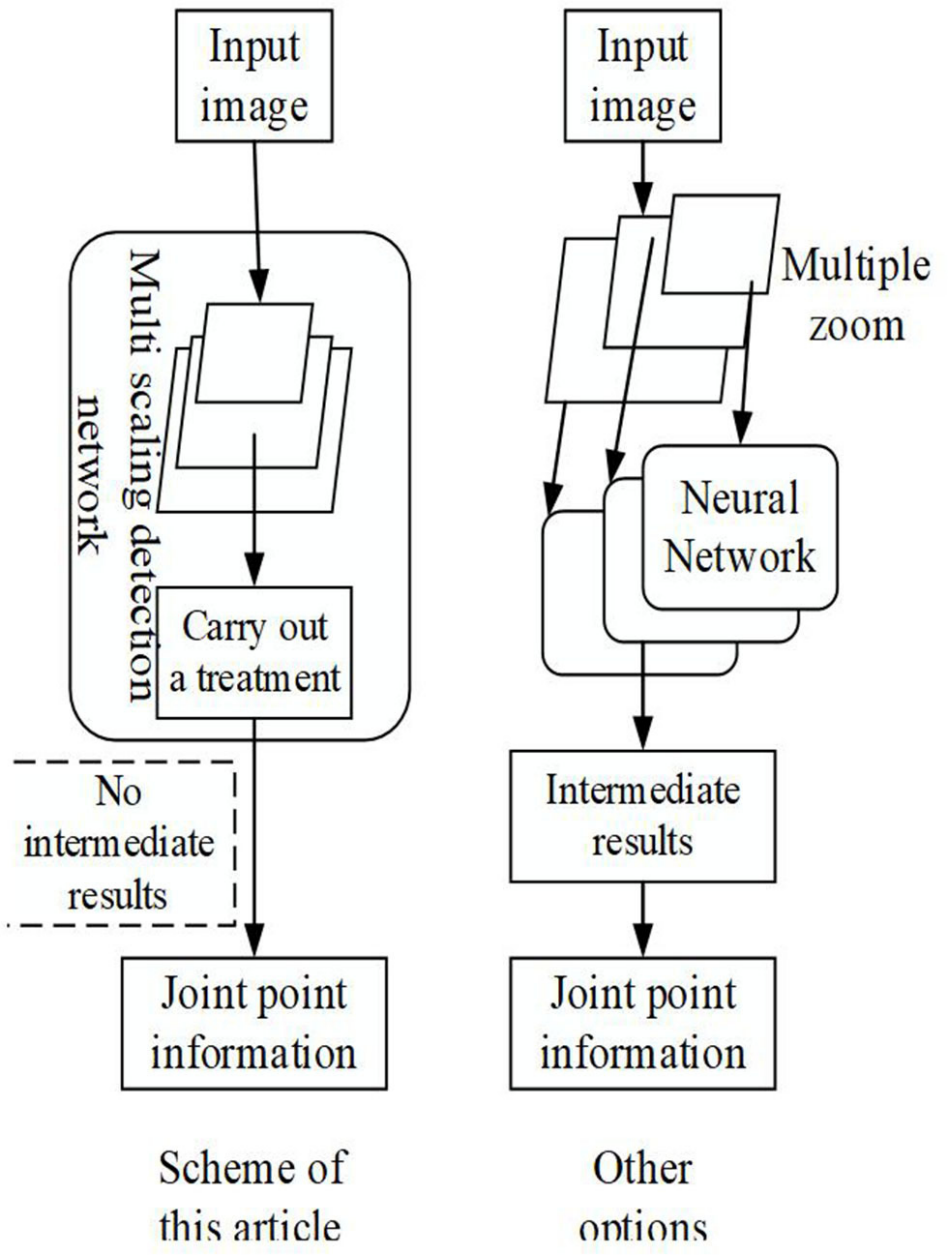
The algorithm framework proposed and the previous scheme.

**Figure 2:**
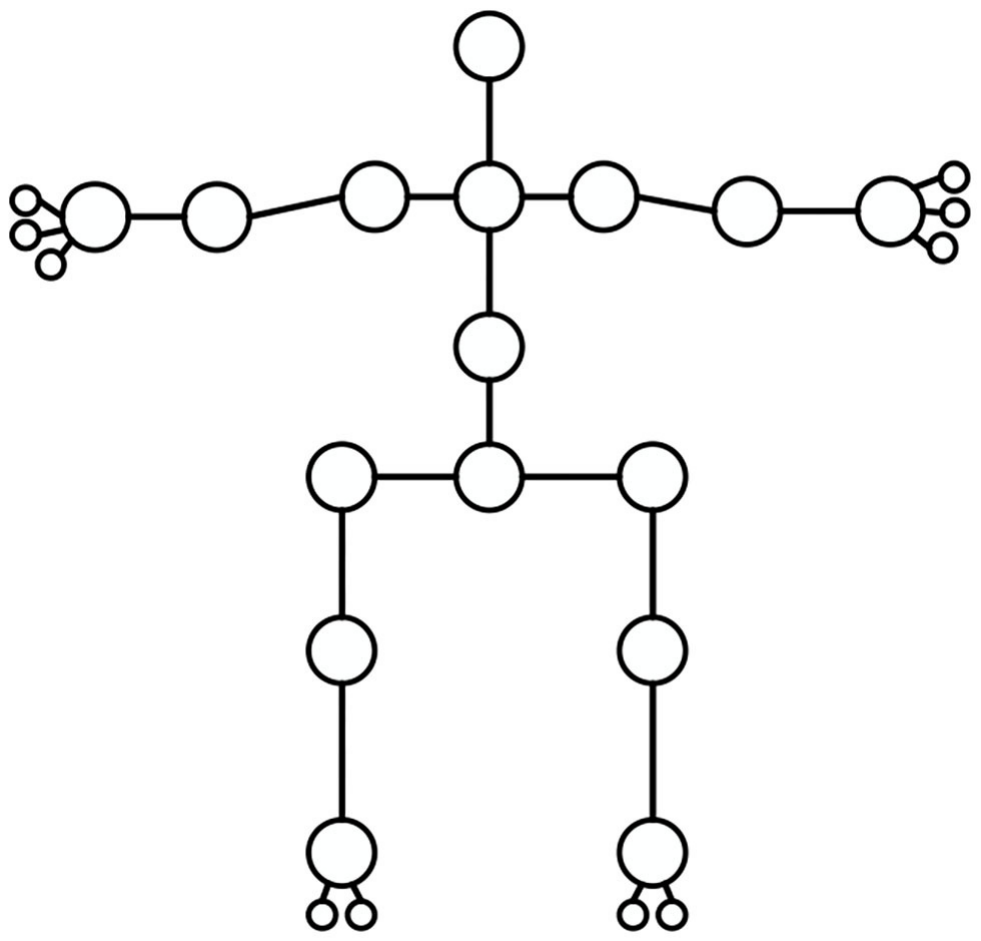
Modeling of human body joints.

Figure 1 suggests that compared with other similar schemes, the scheme proposed can detect multiple scales in one execution without intermediate result representation, achieving the end-to-end and high-speed effects. Feature Pyramid Network (FPN) is adopted here to transform the human body movements in the 2D images into a form suitable for neural network output; it means that the human body movements are regarded as modeling, including multiple joints, as shown in Figure 2.

Each human joint point contains the information about the center 2D coordinates of the joint point, the type of the joint point, and the parent node. Since the full convolutional network (FCN) can only output the feature map, the preceding three kinds of information should be encoded into the feature map. Therefore, for the point (x, y) on the feature graph, the corresponding feature vector is:

(1) $$\begin{array}{r} \left\{P_{C},TargetOffset\right\} \end{array}$$

Among them, *P*_*C*_ represents the probability of the joint point type corresponding to the current position (x, y), while the 2D vector *TargetOffset* represents the relative offset between the coordinates of the parent node and the current joint point.

Therefore, for an input image with given width and height, the network will generate a Heatmap feature map and offset feature map. The feature vector with position (x, y) in Heatmap indicates the joint point type probability corresponding to the current point (x, y). Its value is the following vector:

(2) $$\begin{array}{r} \left\{P_{head},P_{neck},P_{shoulder},P_{arm},P_{hand},P_{pelvis},P_{knee},P_{foot},P_{background}\right\} \end{array}$$

The network using offset position for output is selected instead of directly outputting the memory address of the target joint, because there is no good solution to achieve this goal. The network is required to directly output the offset of the target position, use a simple search to find the corresponding joint points near the target position, and connect them. Based on the above network task design, the corresponding end-to-end network structure is proposed.

ResNet34 is used as the network front-end structure, and the residual structure is adopted. After multiple rounds of convolution, the input image is scaled to a particular size. The deeper network performance can be effectively improved through residual links among layers. On this basis, if each convolutional feature map of CNN is detected separately, the feature pyramid hierarchy used here is generated. The lower-level convolution modules can detect more detailed objects, and the higher-level can detect larger ones. Thus, multiscale problems of the detected objects are well-solved. Meanwhile, the neural network loss function *Loss* is determined as:

(3) $$\begin{array}{r} Loss=Loss_{classification}+Loss_{target} \end{array}$$

(4) $$\begin{array}{r} Loss_{classification}=\overset{height}{\underset{y=0}{\sum }}\overset{width}{\underset{x=0}{\sum }}\overset{9}{\underset{m}{\sum }}{\left(C-Ĉ\right)}^{2} \end{array}$$

(5) $$\begin{array}{r} Loss_{target}=\overset{height}{\underset{j=0}{\sum }}\overset{width}{\underset{i=0}{\sum }}\left({\left(x-\hat{x}\right)}^{2}+{\left(y-ŷ\right)}^{2}\right) \end{array}$$

*Loss*_*classification*_ is the classification loss; *Loss*_*target*_ represents the target position loss; *m* represents the target position point; *x* and *y* are the horizontal and vertical coordinates of the target position point.

The Microsoft COCO dataset is a 2D image dataset provided by Microsoft, which is widely used in tasks such as target detection, motion detection, semantic segmentation, and power segmentation (Ma et al., 2019). It contains information about the human body key points in various scenarios. The COCO action detection dataset contains 120,000 sample pictures, including about 64,000 sample pictures of people. The 2D coordinates of the human body key points are manually labeled for these pictures (Tian et al., 2019). Therefore, the information of this dataset is chosen, and a series of data enhancement strategies is adopted to prevent overfitting, including images' random scaling and cropping, horizontal inversion, and random rotation to a certain angle.

### 3D Human Motion Presentation Based on CNN of DL

A 3D estimation algorithm for body motions with a single camera is proposed based on the DL-based 2D image recognition algorithm for human body motions. Compared with the 2D image recognition algorithm for human body motions, the 3D estimation algorithm for human body motions is much more difficult and complicated (Xu and Schiavone, 2019). A new multitask and multilevel motion estimation neural network algorithm is designed to estimate human postures through the joint positions in 3D space. As RGB images are input, motion joint positions in 2D images, deep information, and connection information are generated. The post-processing system generates all human body poses based on the information. This algorithm's effectiveness is verified by training real-life tasks with virtual datasets.

**Figure 3:**
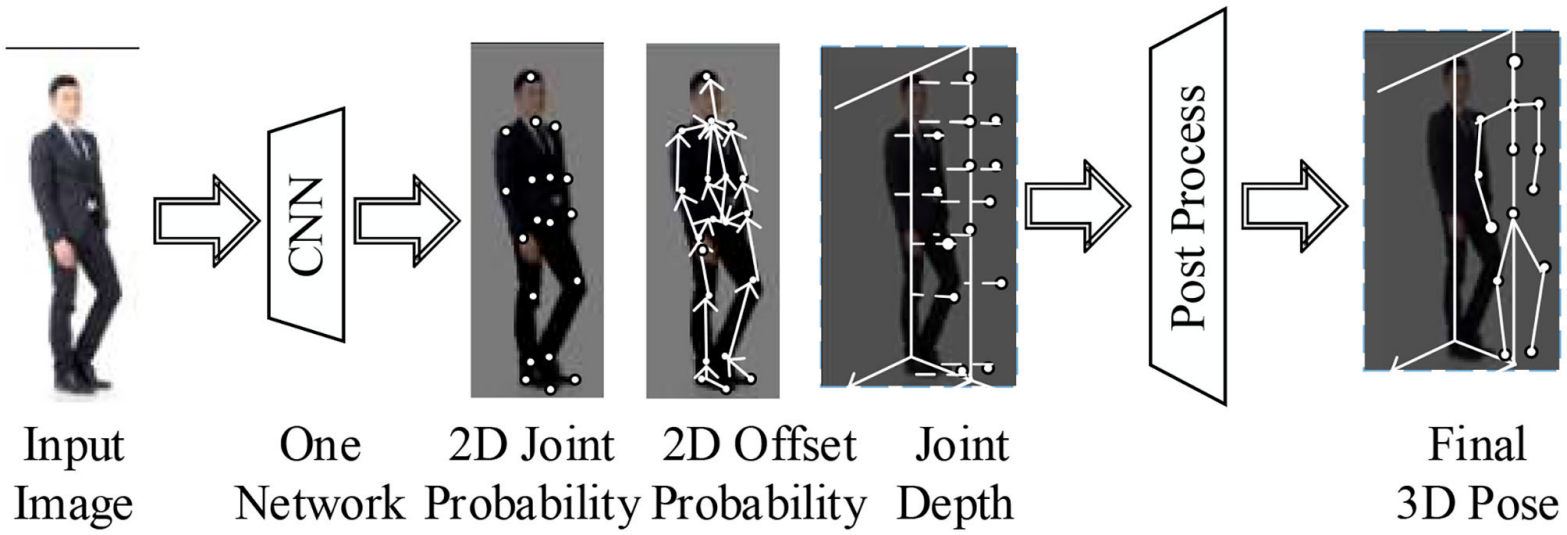
The network structure framework of the 3D estimation algorithm for human body motions.

Figure 3 indicates the neural network algorithm's network structure for multitask and multilevel motion estimation of 3D human body motions. For a given image input, CNN is used for processing. Its three branches can produce three different outputs, and each output encodes a type of information used to reconstruct 3D motions. Quick post-processing reconstructs the final 3D motions through the input of three types of information.

Multitask neural networks are used to output 2D image motion information and 3D stereo information simultaneously (Sadak et al., 2019). A multilevel detection structure is used to maintain speed and accuracy. The second post-processing involves detecting, linking, and automatically matching the poses in the 2D image space and the depth in the 3D space (Shen et al., 2020).

As the 2D image recognition system for human body motions, the 3D human body motion is regarded as a directed graph, in which each joint point corresponds to a human body joint point *M*. Each joint point contains the following information:

(6) $$\begin{array}{r} \left({Class}_{m},X_{m},Y_{m},OffsetX_{m},OffsetY_{m}\right) \end{array}$$

*Class*_m_ donates the type of the current joint point, *X*_m_ and *Y*_m_ represent the current joint point's 2D coordinates, *D*_m_ is the distance between the current joint point and the camera, and *offsetX*_m_ and *offsetY*_m_ are the relative offset from the joint parent point of the current joint point to the current joint point.

From the pose-linking process, the 2D position can be reused. Therefore, depth *D* is the only additional information needed. According to the network output, the post-processing execution map F will convert 2D limb joint points to 3D space in the following ways:

(7) $$\begin{array}{r} F\left(X_{j},Y_{j},D\right)\to \left(X_{3D},Y_{3D},Z_{3D}\right) \end{array}$$

The depth value is encoded as the relative depth of the world space, which is to make the mean value of the probability distribution of the depth value of the joint point as 0 as far as possible, in order to facilitate the learning of the neural network. The reason why relative depth is easier to learn than absolute depth is that it has nothing to do with the position of human body and only needs local information. This is consistent with the characteristics of CNN local receptive field.

The target joint point's position is calculated first to connect the joint points.

(8) $$\begin{array}{r} \left(TargetX_{m},TargetY_{m}\right)=\left(X_{m}+OffsetX_{m},Y_{m}+OffsetY_{m}\right) \end{array}$$

**Figure 4:**
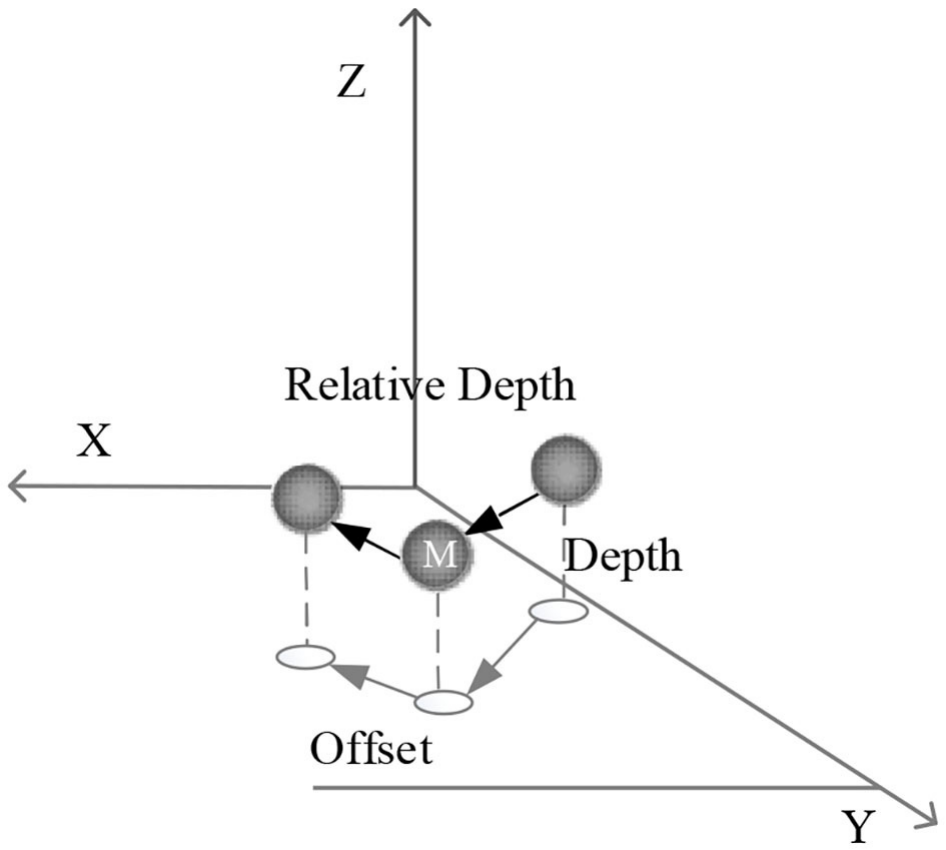
Analysis of 3D joint points.

A circular area with (*TargetX*_*m*_, *TargetY*_*m*_) as the center and R as the radius is searched. If there is a corresponding joint in a correct category in the circle, the current joint is linked to that joint. The search radius is calculated based on the target distance because the farther the parent joint is, the greater the errors are. Figure 4 shows the specific calculation method.

(9) $$\begin{array}{r} l=\sqrt{OffsetX_{m}^{2},OffsetY_{m}^{2}} \end{array}$$

(10) $$\begin{array}{r} R=max\left(l*\alpha +\left(1-\alpha \right),1\right) \end{array}$$

*Scale*_depth_ is quickly calculated and multiplied by *Depth*, so that the correct human joint positions in camera space can be estimated without obtaining camera information.

3D pose-detection dataset rendered by computer graphics is used to assist training, which is an important part of training 3D pose recognition system. Joint Track Auto (JTA) datasets are selected for training. The source of data collection is Rockstar's game Grand Theft Auto 5. It adopts a physics-based rendering architecture, which can produce high-precision character animation. In addition, it contains many images of people with different postures and costumes for use and lots of scenes, and provides a variety of weather.

## Results and Discussion

### Action Recognition Results of 2D Video Images

A series of algorithms in computer vision and image processing is invoked to expedite the research progress and improve the accuracy, and an open source algorithm library is used for some basic algorithms. The Open CV algorithm library is used in the image pre-processing and post-processing stages. The performance and result differences between the proposed scheme and the most widely used Open Pose are compared. The performance of the proposed algorithm and the Open Pose on the GTX 1050 graphics card is 35 and 4.7, respectively, while that on the GTX 1060 graphics card is 58 and 6, respectively. According to the results, in the software environment of CUDA9.2 and Tensorflow-gpu1.10, the performance of the proposed algorithm is 7–9 times higher than that of the most widely used and effective Open Pose under different hardware conditions (Zuixiong, 2020).

Open Pose human posture recognition project is an open source library with Caffe as the framework, which is developed by Carnegie Mellon University (CMU) based on CNN and supervised learning. It can realize the pose estimation of human action, facial expression, finger movement, and so on. It is suitable for single person and multiperson, and has excellent robustness, which is the first real-time multiperson 2D attitude estimation application based on DL in the world. A series of algorithms in the field of computer vision and image processing need to be called. In order to speed up the research progress and accuracy, some basic algorithms are directly called in the form of open source algorithm library. The Open CV algorithm library is called in the image preprocessing and post-processing phase, and the post-processing phase is written in C++ language (Cao et al., 2019).

Adam RMSprop with Nesterov momentum optimizer is used. The initial learning rate is set to 0.002. The adaptive learning rate decay of Keras is utilized. When the training loss function continuously traverses the train set for five times without decreasing, the learning rate is divided by 10.

Table 1 reveals the detection accuracy comparison between this algorithm and the classification algorithm for human body motions in 2D images.

**Table 1:** Detection accuracy comparison between the proposed algorithm and classification algorithm.

	**Network link mapping (%)**	**Classification algorithm (%)**
Accuracy detection	49.8	52.2
Trade-off		2.4

The results prove the detection accuracy comparison between this algorithm and the classification algorithm for human body motions in 2D images in the Microsoft COCO dataset. Although the detection accuracy of the proposed algorithm is 2.4% lower, it has better real-time performance and fewer application scenarios limitations according to the operating performance.

### 3D Animation Presentation

Obtaining high-quality 3D stereo datasets is not easy, which hinders the 3D motion estimation research. One method of dataset capture is to use special clothing with sensors or markers. This type of clothing is often in black with complicated markers, limiting the scope of the characters' dressing.

For the data module, multiple dataset interfaces are designed first. There are two training datasets with different formats, so it is necessary to convert them into the same format through the dataset interface. Then, the Tensor Pack parallel sample generator loads the data of these dataset interfaces and integrates them into the final samples. Python language supports two parallel modes: multithreading and multiple processes. Python multithreading is limited by the global interpreter lock and can only load one bytecode instruction to execute at a time. Multiple processes communication is also very complex. Therefore, Tensor Pack library is selected to assist the parallel design.

The neural network algorithm of multitask and multilevel 3D body motion estimation is evaluated in the test set of the JTA dataset. The RGB images are input and Mean Per Joint Position Error (MPJPE) is used to measure the network performances. Table 2 shows the test results.

**Table 2:** Performance and speed comparison among the proposed algorithm and other schemes.

**Method**		**MPJPE (mm)**	**Speed (ms)**
Open pose	High accuracy	68.7	794.5
	High-speed mode	75.4	138.7
Method of this article		81.2	44.3

First, the 2D detection branch of the MS COCO 2014 dataset training network is used to initialize the network. Then, each time, a sample is randomly selected from the 2D or 3D dataset. It is considered that the use of training methods can make the training process more stable and shorten the training time. Not only 2D datasets or 3D datasets are used, but also a phased and random hybrid training strategy is used. On the one hand, it is hoped to solve the problem of long training time as much as possible. On the other hand, although the latest rendering scheme is used in the JTA dataset to produce high-quality images, such data still cannot be completely equivalent to the actual images taken in real life. Some real images are still needed to improve the generalization ability of the network in real scenes.

Therefore, first, the MS COCO 2014 dataset is used to train the two branches of 2D separately. This is equivalent to training a 2D pose recognition network. Since only one branch participates in the training, the number of parameters and the number of layers of the network are reduced, so the forward propagation and back propagation speed of the network are improved. 50–100 epochs are trained as the initialization of the network.

Next, JTA dataset + MSCOCO dataset are used for joint training. The specific methods are as follows.

1. A set of random numbers with 0-1 distribution is generated, whose length is equal to Batch size.
2. According to 0-1 distribution, loading data from JTA dataset or MSCOCO dataset is selected to form a Batch of training data.
3. For the dataset from MSCOCO, the back propagation of Depth branch is masked.
4. This Batch is added to training.

By using this training strategy, the accuracy of the network in the real pose detection task is successfully maintained, which is 5% higher than using only JTA dataset.

The results suggest that the running speed of the neural network algorithm of multitask and multilevel 3D body motion estimation is 44.3 ms, much lower than the Open Pose's 794.5 and 138.7 ms. Its MPJPE result is 81.2 mm, slightly higher than the Open Pose's 68.7 and 75.4 mm. It indicates that the proposed algorithm is more efficient and convenient in practice.

**Figure 5:**
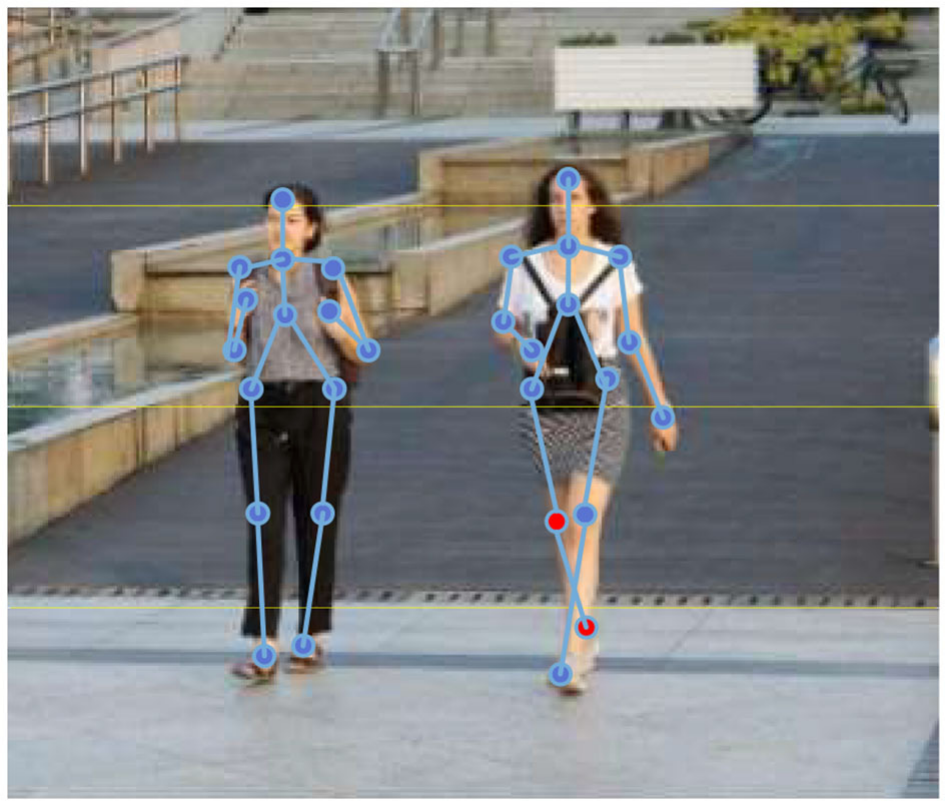
The joint points recognition of the 3D motion in the JTA dataset.

The 3D motion detection dataset rendered by computer graphics is used to assist training, which is a significant part of the 3D human motion system training. Figure 5 shows the 3D human motion recognition in the JTA dataset based on the multi-level-detection neural network framework.

The red joint points in the figure are the covered secondary joint points. The results prove that the proposed algorithm performs excellently with high accuracy recognition in 3D human body motions in the JTA dataset. It contains numerous information, such as people with different actions and in different clothes, and scenes in various weather conditions.

## Conclusion

As AI technology's development, impacts of the DL-based VR technology and the DL technology on modern film and TV works production and audience psychology are studied. New technology developments promote new aesthetics in the era. In film and TV production, audiences have an increasing demand for verisimilitude and immersion of the works, especially in film production. Therefore, a 2D image recognition system for human body motions and a 3D recognition system for human body motions based on the CNN algorithm of DL are proposed here, and an analysis framework is established. The proposed systems are simulated on practical and professional datasets, respectively.

The results show that the computing performance of this algorithm in 2D image recognition is 7–9 times higher than that of the most widely used Open Pose method in software of CUDA9.2 and Tensorflow-gpu1.10. Although the detection accuracy has dropped by 2.4%, it is more efficient and convenient without limitations of scenarios in practical applications. The running speed of the neural network algorithm of multitask and multilevel 3D human body motion estimation is 44.3 ms, much lower than the Open Pose's 794.5 and 138.7 ms. Its MPJPE result is 81.2 mm, slightly higher than the Open Pose's 68.7 mm, and 75.4 mm, but it is more efficient and convenient in practice. Meanwhile, this algorithm performs excellently in the JTA dataset.

Real-time pose recognition and human pose generation technology based on DL are mainly explored. In computer graphics, human posture is extracted, analyzed, and transformed into 3D animation data that can be used in computer programs, and then it is output in real time, which is a very important application in computer graphics.

## Data Availability Statement

The raw data supporting the conclusions of this article will be made available by the authors, without undue reservation.

## Ethics Statement

The studies involving human participants were reviewed and approved by Gachon University Ethics Committee. The patients/participants provided their written informed consent to participate in this study. Written informed consent was obtained from the individual(s) for the publication of any potentially identifiable images or data included in this article.

## Author Contributions

All authors listed have made a substantial, direct and intellectual contribution to the work, and approved it for publication.

## Conflict of Interest

The authors declare that the research was conducted in the absence of any commercial or financial relationships that could be construed as a potential conflict of interest.
